# Bifunctional Tumor-Targeted Bioprobe for Phototheranosis

**DOI:** 10.34133/bmr.0002

**Published:** 2024-01-25

**Authors:** Hae Sang Park, Shinya Yokomizo, Haoran Wang, Sophia Manganiello, Hailey Monaco, Rose McDonnell, Hajin Joanne Kim, Jiyun Rho, Jason Gladstone, Sung Ahn, Harry Jung, Homan Kang, Kai Bao, Satoshi Kashiwagi, Hak Soo Choi

**Affiliations:** ^1^ Gordon Center for Medical Imaging, Department of Radiology, Massachusetts General Hospital and Harvard Medical School, Boston, MA 02114, USA.; ^2^ Department of Otorhinolaryngology-Head and Neck Surgery, College of Medicine, Hallym University, Chuncheon 24253, South Korea.; ^3^ Institute of New Frontier Research Team, Hallym Clinical and Translation Science Institute, Hallym University, Chuncheon 24252, South Korea.

## Abstract

**Background:** Near-infrared (NIR) phototheranostics provide promising noninvasive imaging and treatment for head and neck squamous cell carcinoma (HNSCC), capitalizing on its adjacency to skin or mucosal surfaces. Activated by laser irradiation, targeted NIR fluorophores can selectively eradicate cancer cells, harnessing the power of synergistic photodynamic therapy and photothermal therapy. However, there is a paucity of NIR bioprobes showing tumor-specific targeting and effective phototheranosis without hurting surrounding healthy tissues. **Methods:** We engineered a tumor-specific bifunctional NIR bioprobe designed to precisely target HNSCC and induce phototheranosis using bioconjugation of a cyclic arginine–glycine–aspartic acid (cRGD) motif and zwitterionic polymethine NIR fluorophore. The cytotoxic effects of cRGD-ZW800-PEG were measured by assessing heat and reactive oxygen species (ROS) generation upon an 808-nm laser irradiation. We then determined the in vivo efficacy of cRGD-ZW800-PEG in the FaDu xenograft mouse model of HNSCC, as well as its biodistribution and clearance, using a customized portable NIR imaging system. **Results:** Real-time NIR imaging revealed that intravenously administered cRGD-ZW800-PEG targeted tumors rapidly within 4 h postintravenous injection in tumor-bearing mice. Upon laser irradiation, cRGD-ZW800-PEG produced ROS and heat simultaneously and exhibited synergistic photothermal and photodynamic effects on the tumoral tissue without affecting the neighboring healthy tissues. Importantly, all unbound bioprobes were cleared through renal excretion. **Conclusions:** By harnessing phototheranosis in combination with tailored tumor selectivity, our targeted bioprobe ushers in a promising paradigm in cancer treatment. It promises safer and more efficacious therapeutic avenues against cancer, marking a substantial advancement in the field.

## Introduction

Head and neck squamous cell carcinoma (HNSCC) is the seventh most cause of cancer death worldwide in 2020 (>931,000 new cases and >467,000 deaths) [1]. More than 60% of the patients present with stage III or IV disease at the time of diagnosis, which is characterized by large tumors with marked local invasion and/or regional lymph node metastases [2]. The standard treatment for patients with advanced stage is multimodal approaches, including surgery, radiation, or chemotherapy [3]. The complex anatomy and specialized function of the head-and-neck region dictate that the goals of treating HNSCC are not only to improve the cure rate but also to preserve organ function, such as swallowing, speaking, and respiration [4]. Although several improvements have been accomplished in managing cancer and treatment-related side effects and improving patients’ overall quality of life, a high number of survivors of HNSCC experience treatment-related morbidity causing deficits in physical, social, emotional, and psychological health [5]. Therefore, it is crucial to explore an alternative therapy with less side effects on healthy tissue to improve survival rate as well as overall quality of life [6–8].

Targeted cancer therapy is designed to accumulate in the cancerous tissue while avoiding nonspecific uptake and off-target side effects to normal tissue [7,9–11]. Bioprobes play a crucial role in molecular imaging, allowing for the visualization and detection of specific biological features [12,13]. Phototheranosis is designed to use targeted near-infrared (NIR) bioprobes for cancerous tissue uptake, followed by activating the targeted agents to selectively destroy cancer cells and minimize collateral damage to the normal tissue by using photodynamic therapy (PDT) and/or photothermal therapy (PTT) [14–16]. PDT is designed to produce cytotoxic singlet oxygen (^1^O_2_) through the interaction between optical light and a photosensitizer [17]. However, the short lifetime of ^1^O_2_ in an aqueous solution (0 to 3 μs) and diffusion distance (0 to 20 nm) significantly limits tumor-killing efficacy with PDT, while PTT is another modality using the thermal energy induced by light-to-heat conversion materials to kill cancer cells [18]. A significant downside of this approach is the limited penetration depth of light and photothermal effect, resulting in incomplete tumor ablation. In addition, the excessive temperature may damage the surrounding normal tissue. To overcome such limitations, the combination of PTT and PDT has been investigated since many photosensitizing materials display both photothermal conversion and singlet oxygen generation capacities [16–19].

In the current study, we established a tumor-targeted bifunctional NIR bioprobe using cRGD (cyclic arginine–glycine–aspartic acid)-ZW800-PEG for the real-time imaging and treatment of a xenograft model of HNSCC in mice. Upon intravenous administration, we were able to monitor the biodistribution and cancer targeting of cRGD-ZW800-PEG under real-time NIR imaging. Upon NIR light exposure, intravenously administered cRGD-ZW800-PEG simultaneously produced reactive oxygen species (ROS) and heat and suppressed tumor growth in vivo. This approach represents a new strategy to maximize the therapeutic efficacy of cancer phototheranosis toward complete tumor eradication with minimal local and systemic complications.

## Materials and Methods

### Synthesis of cRGD-ZW800-PEG

All chemicals and solvents were of American Chemical Society or high-performance liquid chromatography (HPLC) purity, purchased from Fisher Scientific (Pittsburgh, PA, USA) or Sigma-Aldrich (St. Louis, MO, USA). Integrin αvβ3-targeted cRGD-ZW800-PEG was synthesized via conventional *N*-hydroxysuccinimide (NHS) ester chemistry as previously described [20]. Briefly, the NHS ester form of ZW800-PEG [21] was conjugated with cyclo(Arg-Gly-Asp-D-Tyr-Lys; cRGD) in the presence of triethylamine in dimethyl sulfoxide. The reaction mixture was purified through facile and efficient solvent purification without the need for column chromatography. The purified product was then analyzed by HPLC (Waters) equipped with simultaneous photodiode array and QDa mass detectors (Waters) and ^1^H-NMR and ^13^C-NMR spectroscopy. All fluorophores showed clear single peaks and high purities prior to in vitro and in vivo studies (>95% purity under the 254 and 750 nm of photodiode array detector).

### Photothermal in vitro studies of cRGD-ZW800-PEG

To elucidate the time-dependent ability for temperature increase, a 500-μl working solution of cRGD-ZW800-PEG at a concentration of 25 μM or phosphate-buffered saline (PBS) was exposed to an 808-nm laser at 200 mW/cm^2^ for 5 min, and thermal images were recorded every minute for each fluorophore using a thermal camera (Xintai HT-19, Donggang, Taiwan).

### Measuring ROS generation of cRGD-ZW800-PEG in a cell-free system

To determine the ROS generation potency of cRGD-ZW800-PEG in response to NIR irradiation, a working solution of cRGD-ZW800-PEG at a concentration of 20 μM in PBS were prepared. Then, 10 μl (0.5 mM) of singlet oxygen sensor green (SOSG) was added to the working solution. The mixed solution was exposed to an 808-nm laser at a light intensity of 30 mW/cm^2^ for 30 min, and fluorescent intensities of SOSG were measured at the fluorescence of 494/525 nm using a BioTek Cytation 5 (Winooski, VT, USA).

### Decomposition analysis of cRGD-ZW800-PEG upon NIR laser irradiation

In order to examine the decomposition of cRGD-ZW800-PEG upon NIR laser irradiation, a 500-μl working solution of cRGD-ZW800-PEG at a concentration of 50 μM in PBS was exposed to an 808-nm laser at 1 W/cm^2^ for 2 to 5 min until the fade of green color. The residual cRGD-ZW800-PEG and the fragments were analyzed by the HPLC-mass spectrometry.

### Theoretical calculations of electron density and minimum energy

Electron density and geometry optimization calculations were performed using Gaussian 16. For these calculations, the density function theory with Becke’s 3-parameter hybrid exchange function with Lee–Yang–Parr gradient-corrected correlation functional (B3LYP function) and 6-31G(d) basis set was used. Solvent effects in water were calculated using the polarizable continuum model method. No constraints to bonds/angles/dihedral angles were applied in the calculations, and all atoms were free to optimize. The Mulliken charge along the heptamethine chain was recorded, after which the formatted checkpoint file (.fchk) was imported into Avogadro 1.2.0. The electron density at isovalue = 0.2 and the electrostatic potential at the surface were calculated and visualized. For the electrical energy of cRGD-ZW800-PEG fragments, energy of 3-dimensional conformations of each fragment was calculated with the Conformer Plugin by MarvinSketch (version 23.2.0) of ChemAxon.

### In vitro cytotoxicity assay of cRGD-ZW800-PEG

Human head and neck cancer FaDu cell line (HTB-43) was purchased from American Type Culture Collection (Manassas, VA, USA) To evaluate the toxicity of cRGD-ZW800-PEG, cells were seeded onto 96-well plates at a density of 400 cells per well in complete Dulbecco’s modified Eagle’s medium (10-013-CV, Corning, USA) with 4.5 g/l glucose, L-glutamine and sodium pyruvate, 10% fetal bovine serum, and 1% penicillin–streptomycin mixture (Lonza, DE17-602E) at 37 °C in 5% CO_2_ humid chamber. Cells were then treated with 1.6, 3.1, 6.3, 12.5, 25, 50, or 100 μM cRGD-ZW800-PEG in the growth media for 24 h at 37 °C. Cell viability was determined using the Cell Counting Kit-8 (CCK-8) from Dojindo Molecular Technologies, Inc. (Kumamoto, Japan). Briefly, 10 μl of CCK-8 was added to each well, and the plates were incubated for 4 h at 37 °C. Absorbance was measured at 450 nm using BioTek Cytation 5. Survival rate was calculated as follows: Survival rate (%) = (*A*_sample_ − *A*_b_)/(*A*_c_ − *A*_b_) × 100, where *A*_sample_, *A*_b_, and *A*_c_ denote the absorbance reading of sample, blanks, and negative control wells, respectively.

### In vitro phototoxicity assay of cRGD-ZW800-PEG

FaDu cells were seeded onto 96-well plates at a density of 400 cells per well. Cells were preincubated for 24 h at 37 °C, followed by treatment with 25 μM cRGD-ZW800-PEG for 24 h. Then, each well was exposed to an 808-nm laser at a power of 100 mW/cm^2^ for 0, 5, 10, or 15 min. After 48 h incubation, 10 μl of CCK-8 was added to each well, and the plates were incubated for 4 h at 37 °C. Absorbance was measured at 450 nm using BioTek Cytation 5.

### In vitro ROS generation from cRGD-ZW800-PEG upon NIR laser irradiation

FaDu cells were seeded into 24-well plates (Corning) at a density of 20,000 cells per well overnight. Cells were then incubated with 25 μM cRGD-ZW800-PEG in complete Dulbecco’s modified Eagle's medium for 24 h. After washing, cells were incubated with 10 μM 2′,7′-dichlorofluorescein diacetate (H_2_DCF-DA) and NucBlue (R37605, Invitrogen, USA) for 30 min. The cells were then washed with Hank’s balanced salt solution and exposed to an 808-nm laser at a light intensity of 100 mW/cm^2^ for 1, 3, or 5 min. The cells were imaged using BioTek Cytation 5. The fluorescence intensity of each cell was determined using ImageJ (version 1.53e, NIH, Bethesda, MD, USA).

### Biodistribution

Eight-week-old athymic nude female mice were purchased from Taconic Farms, Inc (#NCRNU-F, Germantown, NY), and housed in an Assessment and Accreditation of Laboratory Animal Care-certified facility at Massachusetts General Hospital. All animal procedures were performed in accordance with the Public Health Service Policy on Humane Care of Laboratory Animals and approved by the Massachusetts General Hospital Institutional Animal Care and Use Committee (protocol #2016N000136). To determine in vivo biodistribution of cRGD-ZW800-PEG, 100 or 500 nmol of the cRGD-ZW800-PEG was intravenously injected via retro-orbital injection under isoflurane anesthesia into nude mice. For intraoperative NIR fluorescence imaging, we used the K-FLARE imaging system as described previously [11]. In this study, a 760-nm excitation was used at a fluence rate of 4 mW/cm^2^ with white light (400 to 650 nm; 40,000 lux). Animals were then sacrificed after intraoperative NIR fluorescence imaging (24 h postfluorophore injection), and the major organs, including heart, lung, liver, pancreas, spleen, kidney, duodenum, intestine, and abdominal muscle, were excised and imaged for tissue-specific biodistribution. The fluorescence intensity of a region of interest over each organ and muscle was quantified using ImageJ. The signal-to-background ratio (SBR) was calculated as SBR = the average fluorescence intensity of a region of interest / the fluorescence intensity of the muscle.

### Intraoperative fluorescence imaging of a xenograft model of HNSCC in mice

To establish an ectopic HNSCC model, 1 × 10^6^ FaDu cells were suspended in 50 μl of medium/Matrigel (50 v/v%) and were subcutaneously injected into the lower back of nude mice. Real-time fluorescence intensity of the tumor tissue and tumor-to-background ratio (TBR) compared with the surrounding noncancerous tissue were obtained using the FLARE imaging system under anesthesia at 1, 4, 6, and 24 h after intravenous injection of 100 or 500 nmol of cRGD-ZW800-PEG in saline. The TBR was calculated using the following formula: TBR = the fluorescence intensity of the tumor tissue / the fluorescence intensity of the surrounding tissue.

### Histological analysis and immunohistochemistry

To determine the tissue distribution of the cRGD-ZW800-PEG, the tumor tissues from tumor-bearing mice injected with 500 nmol of cRGD-ZW800-PEG were excised and embedded in the Tissue-Tek optimal cutting temperature (OCT) compound (Sakura Finetek, Torrance, CA, USA). Frozen sections were cut at a thickness of 10 μm by a cryostat (Leica, Germany). BioTek Cytation 5 was used for fluorescence imaging. Then, the tissue sections were stained with hematoxylin and eosin (H&E). Bright-field images of H&E-stained slides from a matching field of view were also obtained. For immunohistochemistry of integrin αvβ3, frozen sections were cut at a thickness of 10 μm and the tissue sections were immunostained with primary and appropriate secondary antibodies using avidin-biotin complex/diaminobenzidine histochemistry. The following antibody was used to visualize integrin αvβ3: Integrin Alpha V + Beta 3 polyclonal antibody (50-198-2620; Fisher Scientific, 1:200).

For immunofluorescence analysis of integrin αvβ3 and CD31, FaDu cells-derived tumors were excised when the tumor volume reached around 80 mm^3^. Tumors were embedded in paraffin blocks and sectioned into 10-μm-thick slices. Sectioned tissues were incubated in 0.3% hydrogen peroxide (H_2_O_2_) for 15 min to block endogenous peroxidase activity and then blocked in a 2% horse serum for 60 min. The tissues were incubated with CD31 (303104; BioLegend, San Diego, CA, USA, 1:100) and Integrin Alpha V + Beta 3 polyclonal antibody (50-198-2620; Thermo Fisher Scientific Inc., Waltham, MA, USA, 1:200) at 4 °C overnight and then washed 3 times for 5 min with PBS. Thereafter, the sections were incubated with fluorescent secondary antibody Goat anti-Rabbit IgG (H+L), Alexa Fluor 546 conjugate (A-11035, Thermo Fisher Scientific Inc., Waltham, MA, USA, 1:250), and Donkey anti-mouse IgG (H+L), Alexa Fluor 488 conjugate (A-21202, Thermo Fisher Scientific Inc., Waltham, MA, USA, 1:250) for 120 min at room temperature and then washed. Fluorescent signals were detected using fluorescence microscopy (Carl Zeiss Microscopy GmbH, Jena, Germany).

### Preclinical PTT/PDT therapeutic efficacy with cRGD-ZW800-PEG

FaDu cells were injected into nude mice as described above. When the tumor volume reached around 80 mm^3^, the mice were randomly divided into 4 groups: (a) Control (no treatment), (b) Laser (laser only), (c) cRGD-ZW800-PEG (fluorophore only), and (d) cRGD-ZW800-PEG + Laser group. FaDu tumor mice were intravenously injected with 100 to 500 nmol of cRGD-ZW800-PEG, and the tumors were irradiated with a laser (1.0 W/cm^2^, λ = 808 nm) for 5 to 15 min at 4 h postinjection. Although in vitro data show fast decomposition of cRGD-ZW800-PEG under laser irradiations in 5 min, we applied 15-min laser irradiation for in vivo tumor models to cover 3-dimensional volumetric tumoral tissues. Laser irradiation was performed 3 or 5 times (2- to 3-d intervals). Temperature changes in tumors were monitored using an infrared thermal imaging camera (HT-19). Data were recorded every 1 min throughout the whole laser irradiation process. Time-course NIR fluorescence imaging was conducted using the FLARE imaging system at preinjection, immediately before laser irradiation (4 h postinjection), immediately after laser irradiation, and 24 h postinjection. Tumor growth was monitored using a caliper every 3 d, and the tumor volume (*V*) was measured using the following formula: *V* = 0.5 × longest diameter × (shortest diameter)^2^.

### In vivo ROS generation and TUNEL assay

FaDu tumor-bearing mice were intravenously injected with 500 nmol of cRGD-ZW800-PEG. Thirty minutes later, 25 μl of H_2_DCF-DA (10 μmol/l) was intratumorally injected, followed by laser irradiation (1.0 W/cm^2^, λ = 808 nm) for 5 min. Then, the tumors were harvested and embedded in Tissue-Tek OCT compound without a prefixation step, and the tissue block was frozen at −80 °C. Frozen sections (30 μm thick) were cut by a cryostat (Leica, Germany). These sections were counterstained with 4′,6-diamidino-2-phenylindole. BioTek Cytation 5 was used for DCF-related fluorescence imaging. ROS-positive area in each tumor was quantified using ImageJ. To determine in vivo cytotoxicity effect of cRGD-ZW800-PEG upon laser irradiation, FaDu tumor-bearing mice were intravenously injected with 500 nmol of cRGD-ZW800-PEG. At 4 h postinjection of cRGD-ZW800-PEG, laser irradiation was performed 5 times (2- to 3-d interval). Tumor tissues were harvested 24 h after completion of 5 times of irradiation and then embedded in Tissue-Tek OCT compound without a prefixation step, and the tissue block was frozen at −80°C. Frozen sections (10 μm thick) were cut, and the sections were then subjected to H&E and terminal deoxynucleotidyl transferase (TdT) dUTP nick-end labeling (TUNEL) immunofluorescence staining by iHisto Inc. (Salem, MA, USA). TUNEL-positive area in the fluorescence images of each tumor was quantified using ImageJ.

### Statistical analysis

Statistical analysis was performed using a 1-way analysis of variance (ANOVA) followed by Tukey’s multiple comparisons tests unless otherwise mentioned. *P* values less than 0.05 were considered significant. Results were presented as mean ± standard error of the mean (SEM) for all the analyses. Statistical analysis and curve fitting were performed using Microsoft Excel (Microsoft Inc., Redmond, WA) and Prism version 8 software (GraphPad, San Diego, CA).

## Results

### ROS and heat generation of cRGD-ZW800-PEG

**Fig. 1.**
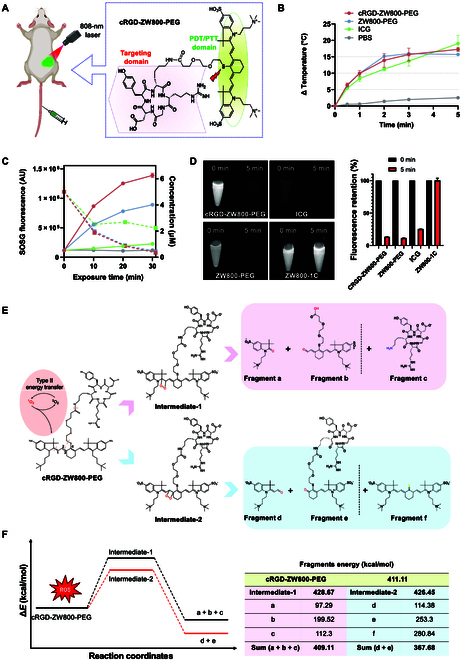
Chemical structure, photothermal and photodynamic effects, and decomposition analysis of cRGD-ZW800-PEG. (A) Chemical structure composed of targeting and PDT/PTT domains. (B) Temperature changes of each fluorophore at 25 μM in PBS upon 808-nm laser irradiation at 200 mW/cm^2^ over 5 min. (C) Measurements of singlet oxygen (^1^O_2_) generation of each fluorophore at 20 μM in PBS upon 808-nm laser irradiation at 30 mW/cm^2^ over 30 min using SOSG (*n* = 3, mean ± SEM). (D) Decomposition of cRGD-ZW800-PEG and ZW800-PEG upon 808-nm laser irradiation at 1.0 mW/cm^2^ for 5 min. ICG and ZW800-1C were applied as the positive and negative controls, respectively. (E) Photodegradation of cRGD-ZW800-PEG and its nonfluorescent keto fragments a to f confirmed by HPLC-MS analysis, and its potential mechanism of action (F).

To confer cancer targetability, we first conjugated a cancer-targeting cRGDyK motif to ZW800-PEG via conventional NHS ester chemistry as previously established [20] (Fig. 1A). The sulfide group on the *meso*-carbon position of ZW800-PEG contributes to the maximum emission wavelength shift to 800 nm, enabling PTT [21]. To determine heat generation of cRGD-ZW800-PEG upon NIR light exposure, we measured the temperature changes on a test tube containing conjugated or unconjugated ZW800-PEG or indocyanine green (ICG) upon 808-nm laser irradiation. All 3 fluorophores showed consistent temperature increases within the first 3 min. ZW800-PEG and cRGD-ZW800-PEG reached a plateau over 5 min of laser irradiation, while ICG consistently emitted heat upon laser irradiation (Fig. 1B).

Following the temperature result, we further hypothesized that cRGD-ZW800-PEG would be decomposed upon NIR light exposure and release ^1^O_2_ due to the oxidation-sensitive thiol linker on the *meso*-carbon [22]. As shown in Fig. 1C, cRGD-ZW800-PEG showed the steepest increase of SOSG fluorescence signal among all the tested fluorophores upon NIR light irradiation. Interestingly, the fluorescence signal of cRGD-ZW800-PEG was higher compared with ZW800-PEG, implying that the conjugation of cRGD moiety has a positive impact on ^1^O_2_ generation. The concentration of cRGD-ZW800-PEG or ZW800-PEG decreased over the irradiation period, and it showed a faster decrease than ICG (Fig. 1C), indicating that cRGD-ZW800-PEG was rapidly decomposed upon NIR irradiation. Similar to ZW800-PEG, the overall electron density on the heptamethine chain of cRGD-ZW800-PEG increases due to the π-donation from the sulfur atom, which makes the heptamethine chain easier to undergo the oxidative addition with reactive singlet oxygen species [23,24].

In order to confirm the involvement of such a mechanism, we analyzed the decomposition products of cRGD-ZW800-PEG. Due to the relatively higher electron densities on the heptamethine chains, ICG, ZW800-PEG, and cRGD-ZW800-PEG showed faster decompositions (>75%) under laser irradiations, compared to ZW800-1C that kept almost the unreduced fluorescence intensity and stability (Fig. 1D). As shown in Fig. 1E and Fig. S1, upon laser irradiation, cRGD-ZW800-PEG yielded nonfluorescent keto fragments within 5 min [22–24]. The residual cRGD-ZW800-PEG and the fragments a**** to e**** were analyzed and confirmed by HPLC-MS analysis (Fig. 1E). Overall, the decomposition process of cRGD-ZW800-PEG was exothermic in the 2 possible paths (Fig. 1F) [25,26]. As expected, we found that fragments ****a, b, d, and ****e were generated through the oxidative addition to the reactive singlet oxygen species. ICG and ZW800-PEG showed similar paths of decomposition, where the breakdown of heptamethine chains happened between C1’ and C2’. Surprisingly, we also found the breakage of the amide and thioether bonds of cRGD-ZW800-PEG (fragments c and f), which are considered to be resistant to oxidative stress but fragile to cathepsin B in the tumor microenvironment (TME) [27]. Compared to the traditional and recyclable PTT effects (S1 to S0, vibration relaxation), the exothermic process of cRGD-ZW800-PEG upon laser irradiation is irreversible, plus the decompositions of the heptamethine chains and nearby chemical bonds.

### In vitro phototherapeutic efficacy of cRGD-ZW800-PEG

**Fig. 2.**
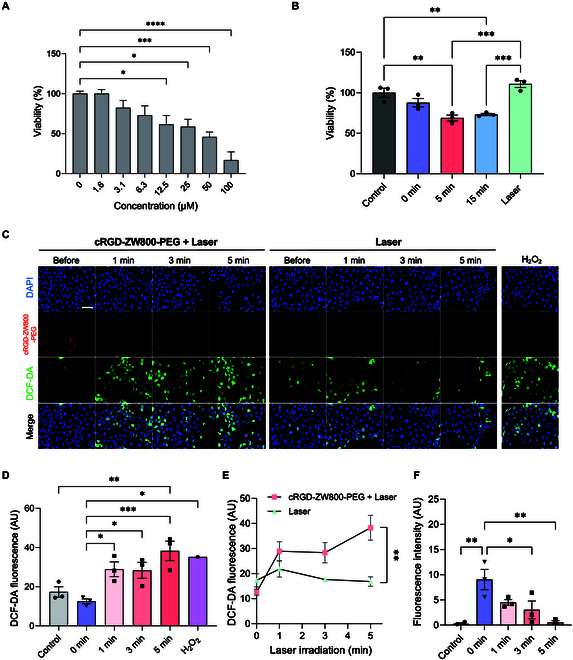
Cytotoxicity and in vitro phototherapeutic effects of cRGD-ZW800-PEG. (A) Cytotoxicity of cRGD-ZW800-PEG. FaDu cells were treated with 1 to 100 μM of the fluorophore for 24 h, followed by an assessment of cell viability. (B) In vitro cell viability upon NIR-laser with cRGD-ZW800-PEG. Cultured FaDu cells were treated with 25 μM cRGD-ZW800-PEG for 24 h, followed by 100 mW/cm^2^ laser irradiation for 0 to 15 min. (C to E) Measurements of ROS generation upon photon-induced therapy. FaDu cells were treated with 25 μM cRGD-ZW800-PEG for 24 h, followed by loading 10 μM H_2_DCF-DA, then exposed to an 808-nm laser at an irradiance of 100 mW/cm^2^ for 1 to 5 min. H_2_O_2_ alone served as the positive control. (C) Representative fluorescence images of cells. Scale bar = 100 μm. (D) Quantification of the DCF-fluorescence intensity. (E) Comparison of ROS generation upon NIR laser irradiation with or without cRGD-ZW800-PEG. (F) Quantification of the concentration of cRGD-ZW800-PEG during the laser irradiation. Statistical analysis was performed by 2-way ANOVA followed by Tukey’s multiple comparisons. *P < 0.05, **P < 0.01, ***P < 0.001 (*n* = 3, mean ± SEM). DAPI, 4′,6-diamidino-2-phenylindole.

Since ^1^O_2_ and heat generations are key elements in PDT and PTT, respectively [28], we next hypothesized that cRGD-ZW800-PEG would show cytotoxic effects on cancer cells upon NIR light irradiation. First, we investigated the cytotoxicity of cRGD-ZW800-PEG without laser irradiation. Cultured FaDu cells were incubated with various concentrations of cRGD-ZW800-PEG. The viability measurement results indicated that cRGD-ZW800-PEG was cytotoxic against the cells at concentrations higher than 12.5 μM (Fig. 2A), showing that cRGD-ZW800-PEG has little cytotoxicity on its own. Next, in vitro phototherapeutic efficacy was evaluated in FaDu cells with various laser exposure times. To facilitate an efficient comparison, we established a baseline condition where 60% viability of 25 μM cRGD-ZW800-PEG-treated cells is considered as 100%. While laser irradiation alone showed no effect, 5 and 15 min of laser irradiation substantially decreased cell viability compared to no laser irradiation control in the presence of cRGD-ZW800-PEG exhibiting 68.8% and 73.2% viability, respectively (Fig. 2B). These results indicated that cRGD-ZW800-PEG, in combination with NIR light irradiation, shows cytotoxic effects on FaDu cells, which supported further in vivo exploration of the therapeutic effect of cRGD-ZW800-PEG.

HNSCC targetability of cRGD-ZW800-PEG was confirmed in vitro by fluorescence microscopy using FaDu cells. cRGD-ZW800-PEG showed high uptake in FaDu cells (Fig. 2C). Our finding is consistent with the literature showing that cRGD moiety actively accumulates in HNSCC cells due to the recognition of various integrins, including αvβ6 and αvβ5 as well as αvβ3 overexpressed on the malignant cell surface [29,30]. Next, the photodynamic response of cRGD-ZW800-PEG was evaluated by measuring the generation of ^1^O_2_ under various laser exposure times using H_2_DCF-DA (Fig. 2C), which detects ROS including ^1^O_2_ [31]. Upon NIR laser irradiation of FaDu cells incubated with cRGD-ZW800-PEG, ROS-reacted DCF-dependent fluorescence signal significantly increased (Fig. 2C and D, 5 min versus no laser control: ***P <0.001). DCF-dependent fluorescence signal kept increasing over time, but NIR laser irradiation alone did not affect the fluorescence signal, indicating that ROS was derived from the interaction between NIR laser and cRGD-ZW800-PEG (Fig. 2E). Consistently, the concentration of cRGD-ZW800-PEG in FaDu cells exhibited a steady decrease over the exposure time, which was 0.497-, 0.333-, and 0.005-fold decrease compared to the no laser control (Fig. 2C and F), indicating that cRGD-ZW800-PEG underwent decomposition upon NIR laser irradiation.

### In vivo evaluation of tumor cell targetability and biodistribution of cRGD-ZW800-PEG

**Fig. 3.**
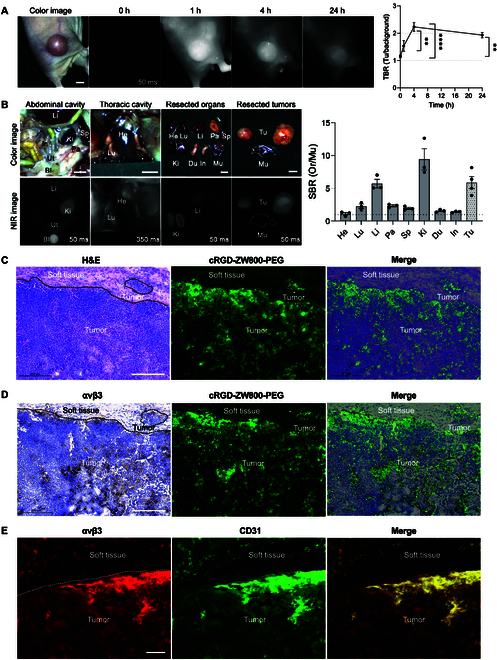
In vivo evaluation of tumor targetability and biodistribution of cRGD-ZW800-PEG. cRGD-ZW800-PEG (500 nmol) was injected into a murine xenograft model of HNSCC. (A) Quantitative time-course assessment up to 24 h postinjection (*n* = 7, mean ± SEM; **P < 0.01, ****P < 0.0001). Scale bars = 5 mm. (B) Biodistribution and SBR of resected organs and tumors. SBR was determined by comparing the signals of major organs against muscle (*n* = 3, mean ± SEM). Scale bar = 5 mm. Ga, gallbladder; He, heart; Lu, lungs; Li, liver; Pa, pancreas; Sp, spleen; Ki, kidneys; Du, duodenum; In, intestine; Mu, muscle; Ga, gallbladder; Bl, bladder; Tu, tumor. (C) Histological and (D) immunohistochemical analyses of integrin αvβ3 in the tumor. The tumor was sectioned 4 h postinjection, imaged on the fluorescence microscopy, and then stained with an anti-αvβ3 antibody, followed by an appropriate secondary antibody using avidin-biotin complex/diaminobenzidine histochemistry. Scale bar = 200 μm. (E) Immunofluorescence staining of integrin αvβ3 and CD31 in the tumor. Tumors were excised when the tumor volume reached around 80 mm^3^ and stained with anti-αvβ3 and anti-CD31 antibodies, followed by appropriate secondary antibodies. Scale bar = 50 μm.

The in vivo targetability cRGD-ZW800-PEG was determined in a murine xenograft model of HNSCC. First, 500 nmol of cRGD-ZW800-PEG was administered to tumor-bearing mice, followed by intravital NIR imaging 1 to 24 h after injection and ex vivo tissue biodistribution 24 h after injection. The quantitative time-course assessment revealed that TBR increased up to 4 h postinjection (2.2) and then washed out until 24 h postinjection (1.9) (Fig. 3A). This result indicates that 4 h postinjection is the optimal time point for laser treatment with maximal effect on cancer and minimal adverse effect on surrounding normal tissue. Then, the biodistribution study showed a high fluorescence signal in the kidney, indicating that renal clearance is the primary excretion pathway for cRGD-ZW800-PEG (Fig. 3B), which is consistent with our previous study [20]. A notable fluorescence signal was observed in the liver, suggesting cRGD uptake in the integrin-expressed hepatocytes [32]. The excised tumor tissue showed a high SBR of the tumor (SBR = 5.9), confirming excellent tumor targeting with cRGD-ZW800-PEG.

Histologically, strong cRGD-ZW800-PEG fluorescence signals were detected throughout the tumor tissue with no substantial fluorescence signals in surrounding normal tissues (Fig. 3C). Small tumor nest embedded in normal tissue was also efficiently detected by fluorescence signal. Immunohistochemistry analysis confirmed the expression of integrin αvβ3 in the tumor tissue, and the fluorescence intensity of cRGD-ZW800-PEG was positively colocalized with integrin αvβ3 expression (Fig. 3D). As confirmed by the in vitro study above, although FaDu cells are negative for integrin αvβ3 expression, RGD sequence shows affinity to the angiogenic tumor tissue and can detect αvβ3-positive intratumoral HNSCC [29,33]. Colocalization between integrin αvβ3 and CD31 within the tumor tissue was also confirmed in immunofluorescence staining (Fig. 3E). These results demonstrate that cRGD-ZW800-PEG can target the TME, including tumor vasculature. Disrupting the tumor vessels is an established strategy to starve tumor cells by inhibiting the nutrients supplement for tumor growth [34]. In particular, Gao et al. [35] developed a PTT using a NIR-activated “nanobomb”, which was fabricated via the encapsulation of vinyl azide into cRGDfE peptide-functionalized, hollow copper sulfide nanoparticles. Their results demonstrated that PTT could be used to disrupt tumor vessels, thereby interrupting the supplement of nutrients and eventually inhibiting tumor growth.

### In vivo NIR phototherapeutic efficacy of cRGD-ZW800-PEG

**Fig. 4.**
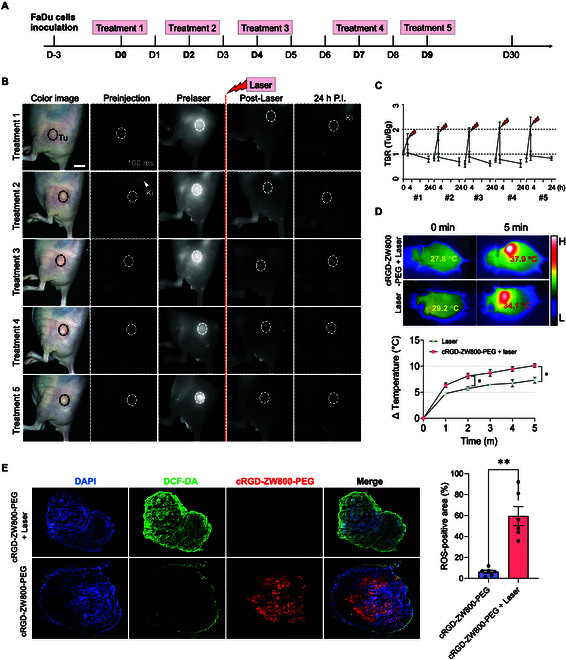
In vivo evaluation of cRGD-ZW800-PEG decomposition under 808-nm laser irradiation. (A) A schematic of the laser treatment. Tumors were irradiated by an 808-nm laser (1.0 W/cm^2^) for 5 min 4 h postinjection of cRGD-ZW800-PEG in tumor-bearing mice. A total of 5 times of laser irradiation was performed on the same site at intervals of 2 to 3 d. (B) Representative time-course NIR images during the laser treatment. NIR fluorescence imaging was conducted using the FLARE imaging system at preinjection, prelaser (4 h postinjection of cRGD-ZW800-PEG), postlaser (immediate after laser irradiation), and post-24 h injection of cRGD-ZW800-PEG. Tu, tumor. Scale bar = 5 mm. (C) Quantitative time-course assessment of TBR. TBR was observed at preinjection, prelaser (4 h postinjection of cRGD-ZW800-PEG), postlaser (immediate after laser irradiation), and post-24 h injection of cRGD-ZW800-PEG by NIR imaging (*n* = 4, mean ± SEM). (D) Temperature changes of the irradiated tumor in each group (*n* = 6 each, mean ± SEM). (E) Fluorescence images and quantitative measurement of ROS from in vivo tumors treated with cRGD-ZW800-PEG with or without laser irradiation. Scale bar = 1,000 μm (cRGD-ZW800-PEG + Laser), and 2,000 μm (cRGD-ZW800-PEG). *P < 0.05; **P < 0.01 by 2-way ANOVA.

Next, cRGD-ZW800-PEG was evaluated in the xenograft model of HNSCC in mice to investigate its in vivo phototheranostic performance. Four hours after injection with 500 nmol of cRGD-ZW800-PEG, the tumors were irradiated by an 808-nm laser at an irradiance of 1.0 W/cm^2^ for 5 min. Control groups included laser treatment only, cRGD-ZW800-PEG only, and no treatment. A total of 5 times of laser irradiation was performed on the same site at intervals of 2 to 3 d (Fig. 4A). In order to determine the in vivo decomposition of cRGD-ZW800-PEG under the laser irradiation, intravital NIR imaging at pre- and postlaser irradiation was performed. As shown in Fig. 4B and C, TBRs were around 2.0 prior to the laser treatment, which decreased remarkably after 5 min of laser irradiation (TBR <1.0), indicating that cRGD-ZW800-PEG was completely decomposed by laser irradiation. Furthermore, the tumor targetability and decomposition of cRGD-ZW800-PEG upon laser irradiation remained constant even after multiple laser irradiations, which indicates that therapeutic resistance was not developed by multiple irradiations in the current treatment protocol.

We further determined the thermal responses of cRGD-ZW800-PEG in the tumor tissue upon laser irradiation in vivo. Thermal images recorded during laser irradiation show that the temperature of the tumor region in the cRGD-ZW800-PEG + Laser group exhibited an elevation of temperature of approximately 10 °C (Fig. 4D). On the other hand, the increase in temperature in the laser-only group was less than 7 °C. The temperature in cRGD-ZW800-PEG + Laser group was significantly higher at 2 and 5 min than the Laser group (cRGD-ZW800-PEG + Laser versus Laser: *P < 0.05 at 2 or 5 min). cRGD-ZW800-PEG + Laser group showed a rapid temperature increase within the first 2 min upon irradiation, whereas Laser only group showed slow and sustainable temperature change over 5 min of irradiation. The temperature changes in the normal tissue were observed to be less than 10 °C during the measurement (<43 °C), under the established thermal damage threshold field [36,37].

**Fig. 5.**
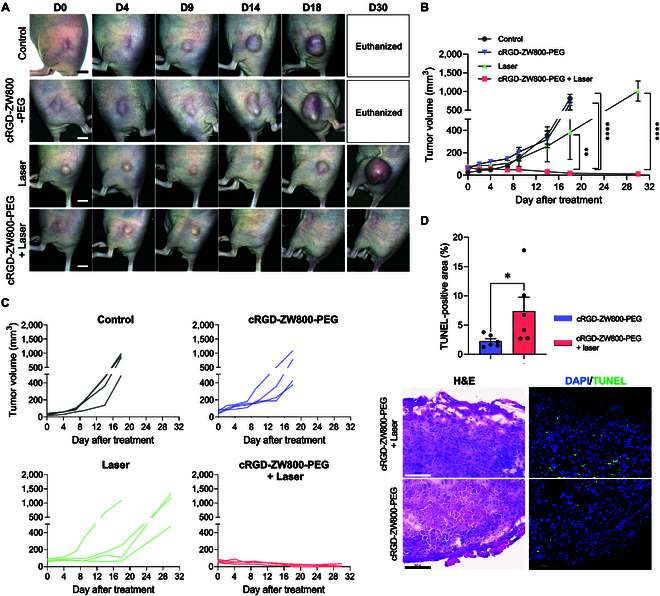
In vivo phototherapeutic efficacy of cRGD-ZW800-PEG. (A) Representative photos of the xenograft tumor in the mouse back over 30 d after the initiation of the treatments. Tumor-bearing mice were injected with 500-nmol cRGD-ZW800-PEG and irradiated with an 808-nm laser (1.0 W/cm^2^) for 5 min every 2 to 3 d (5 times in total). The control group includes no treatment, laser only, and fluorophore only. Scale bar = 5 mm. (B) Average and (C) individual growth curves of the tumors (*n* = 4, mean ± SEM). (D) Evaluation of cytotoxicity of the photon-induced therapy. Twenty-four hours after the completion of the final treatment, the tumors were sampled, sectioned, and then subjected to TUNEL assay. (Left) H&E and (right) TUNEL staining images quantification of TUNEL-positive area in each group are shown. *P < 0.05, **P < 0.01, ****P < 0.0001 by 2-way ANOVA. Scale bar = 100 μm.

To determine in vivo PDT response, we further investigated ROS generation from cRGD-ZW800-PEG in the tumor in the xenograft model. To this end, ROS-sensitive H_2_DCF-DA was intratumorally injected into the tumor sites following injection with cRGD-ZW800-PEG into the xenograft model. The tumors were then exposed to an 808-nm laser. Analysis of DCF fluorescence in the frozen tumor samples revealed that ROS were widely distributed in the postirradiated tumors (Fig. 5D). DCF-fluorescence-positive area was significantly higher in the cRGD-ZW800-PEG + Laser group than that of the cRGD-ZW800-PEG-only group (Fig. 4E; **P < 0.01). While the cRGD-ZW800-PEG + Laser group showed almost no fluorescence signal of cRGD-ZW800-PEG in the tumor tissue, the strong fluorescence signal of cRGD-ZW800-PEG remained throughout the tumor in the cRGD-ZW800-PEG-only group without laser irradiation, indicating the rapid decomposition of cRGD-ZW800-PEG and ROS generation in the tumor tissue upon laser irradiation. These results consistently demonstrate that the laser irradiation causes the generation of heat and ^1^O_2_ followed by the decomposition of cRGD-ZW800-PEG, suggesting that cRGD-ZW800-PEG could be used as a phototherapeutic for PDT and PTT.

Next, the therapeutic efficacy of cRGD-ZW800-PEG was evaluated in the xenograft model using the same in vivo treatment protocol established above. To determine an optimal therapeutic dose of cRGD-ZW800-PEG, in vivo phototheranostic performance of 100, 250, and 500 nmol of cRGD-ZW800-PEG was evaluated in the xenograft mice model of HNSCC. Four hours after injection, the tumors were irradiated by an 808-nm laser at an irradiance of 1.0 W/cm^2^ for 5 min. As shown in Fig. S2A to C, TBRs in each group were around 2.0 at 4 h after fluorophore injection (0 min laser treatment), and decreased abruptly after 5 min of laser irradiation. Temperature changes of the irradiated tumor showed a temperature increase (< 10 °C) with 100 and 250 nmol of cRGD-ZW800-PEG. On the contrary, a sufficient temperature increase (≈10 °C) was observed with 500 nmol to achieve the cytotoxic and heat-shock effect (Fig. S2D) [38,39]. Based on this result, we advanced a dose of 500 nmol to the next stage.

We also determined an optimal therapeutic schedule for phototheranosis. Tumors were irradiated by an 808-nm laser at an irradiance of 1.0 W/cm^2^ for 5 min at 4 h postinjection of cRGD-ZW800-PEG (100 nmol). A total of 3 times of laser irradiation was performed on the same site at intervals of 2 d (Fig. S3A). The tumor growth of cRGD-ZW800-PEG injected mice was marginally faster than the control, indicating insufficient therapeutic effect with suboptimal PTT effect with this regimen and potential tumor promotion effect with mild heat generation and NIR laser exposure (Fig. S3B) [40]. In contrast, upon 3-time irradiations with 500 nmol, tumors in the cRGD-ZW800-PEG + Laser group exhibited significant growth delay compared to the control group (Fig. S3C). However, complete tumor eradication was not achieved in this regimen.

Based on these results, we employed 5-time irradiation regimen with 500 nmol of cRGD-ZW800-PEG. As shown in Fig. 5A to C, cRGD-ZW800-PEG + Laser group showed the most pronounced tumor growth delay compared to the control groups. Furthermore, tumor regrowth was not observed during the experimental period, indicating that the combination of cRGD-ZW800-PEG and NIR laser irradiation could achieve complete control. In addition, no observable skin lesions or signs of inflammation suggestive of thermal damage were present in the tumor site subjected to laser irradiation. The tumors in no treatment or cRGD-ZW800-PEG only group showed a similar growth rate, indicating less efficient tumor suppression. Interestingly, the Laser only group could suppress tumor growth during the course of the multiple laser irradiation [40]. However, the tumor regained its progression after the completion of 5 times irradiation, suggesting that the laser alone was insufficient to control tumor progression. To validate the cytotoxic effect of cRGD-ZW800-PEG followed by laser irradiation, we performed a TUNEL assay on the tumor tissue 24 h after the completion of 5 times irradiation. TUNEL-positive area was significantly higher in the cRGD-ZW800-PEG + Laser group compared to the cRGD-ZW800-PEG only group (Fig. 5D). These results indicate that cRGD-ZW800-PEG with NIR laser irradiation shows the cytotoxic effect to induce tumor cell apoptosis.

## Discussion

Our study provides compelling evidence for the efficacy and specificity of cRGD-ZW800-PEG as a potent agent for NIR phototheranostics in the context of HNSCC. Notably, cRGD-ZW800-PEG exhibits both heat generation and release of ROS upon NIR irradiation, resulting in a combined PDT and PTT effect. This functionality maximizes the therapeutic efficacy in suppressing tumor growth, followed by the photodegradation of the injected dose. We observed that cRGD-ZW800-PEG exhibited a rapid and substantial increase in temperature when subjected to an 808-nm laser, reaching a plateau within 5 min. The temperature changes in cRGD-ZW800-PEG were accompanied by ROS generation due to the photosensitization mechanisms of ZW800-PEG [21], further supporting its potential for PDT. This combination of PDT and PTT modes of action contributed to the effective eradication of cancer cells. An intriguing feature of cRGD-ZW800-PEG is, in contrast to ICG, its ability to generate heat while maintaining a temperature below the established threshold for thermal damage [36,37], enabling effective PTT while ensuring the safety of surrounding normal tissue. This suggests that the photothermal effects of cRGD-ZW800-PEG were carefully controlled to minimize the impact on surrounding healthy tissues. Furthermore, the decomposition of tumor-targeted bioprobe upon laser irradiation was confirmed, highlighting its irreversible exothermic process.

Our study also highlighted the excellent targetability of cRGD-ZW800-PEG to HNSCC cells both in vitro and in vivo. cRGD-ZW800-PEG demonstrates rapid and specific accumulation within tumor tissue within 4 h postinjection, with minimal uptake observed in normal tissue. This finding is consistent with the literature showing that cRGD moiety targets various integrins, including αvβ6 and αvβ5 as well as αvβ3 overexpressed on HNSCC cell surface [29,33]. This targeted accumulation mitigates the risk of unintended damage to healthy tissue. Furthermore, the biodistribution analysis indicated that cRGD-ZW800-PEG is primarily cleared through renal excretion, with notable uptake in the liver due to integrin expression in hepatocytes [32]. This data suggests the potential for cRGD-ZW800-PEG to target integrin-expressing cancer cells in various organs.

Our findings hold significant implications for the development of targeted cancer therapies, particularly in the treatment of HNSCC. The ability to selectively target cancer cells while sparing neighboring healthy tissue is a crucial advancement in cancer treatment. This targeted approach has the potential to minimize collateral damage and reduce side effects associated with conventional therapies. Moreover, the effectiveness of cRGD-ZW800-PEG in inducing tumor cell apoptosis underscores its potential as a promising therapeutic agent. Additionally, in contrast to nanoparticles and antibodies, the small molecular weight of cRGD-ZW800-PEG can rapidly distribute in their targets, and unbound molecules can be rapidly excreted [41,42] and are amenable to rapid, economical, and large-scale production. These findings highlight the promising potential of cRGD-ZW800-PEG as a multifunctional agent for noninvasive imaging, targeted cancer therapy, offering specific tumor accumulation, and safety considerations.

While our study presents promising results regarding the efficacy of cRGD-ZW800-PEG as a phototheranostic agent for HNSCC, several limitations should be considered. (a) Immune response and clearance: The rapid decomposition of cRGD-ZW800-PEG upon NIR laser irradiation may lead to the release of degradation products. These breakdown products could potentially trigger an immune response, impacting the overall safety and tolerability of the treatment. We are currently conducting comprehensive immunological studies, including investigations into potential immunogenicity and long-term effects, which are essential before clinical translation. (b) Tumor heterogeneity and targetability: HNSCC, like many other cancers, is known for its heterogeneity, both at the cellular and molecular levels [29,33]. In addition, not all HNSCC tumors exhibit the same level of integrin expression, which could impact the effectiveness of this treatment in certain cases. While our study demonstrated excellent targetability of cRGD-ZW800-PEG to HNSCC cells, it is important to acknowledge the potential variability in integrin expression among different patients and tumor types. This heterogeneity can affect targeting and treatment response. (c) Optimal treatment parameters: Our study identified an optimal treatment regimen involving multiple laser irradiations. However, the determination of the precise treatment parameters, such as the number and duration of laser sessions, may require further optimization. The most effective treatment regimen could vary depending on factors such as tumor size, location, and individual patient characteristics. Overcoming these challenges is crucial for advancing the development of targeted, safe, and effective therapies for HNSCC and potentially other cancers.

In summary, our study demonstrates that cRGD-ZW800-PEG is a potent NIR bioprobe capable of noninvasive imaging and targeted cancer cell destruction through the synergistic effects of PDT and PTT. By harnessing specific light wavelengths irradiated by a low-power laser, the bioprobe can undergo controlled photodegradation, resulting in the release of cytotoxic agents or the disruption of crucial cellular processes within the tumor microenvironment. This approach provides a precise and localized intervention that has the potential to effectively impede tumor progression while minimizing harm to healthy tissues. Importantly, all unbound molecules were excreted renally. The excellent targetability, specificity, and therapeutic efficacy of cRGD-ZW800-PEG make it a promising candidate for further development as a phototheranostic agent for HNSCC and potentially other cancers. Further research and clinical investigations are warranted to explore its full potential in the clinical setting.

## Data Availability

The datasets and materials used and/or analyzed during the current study are available from the corresponding author upon reasonable request.

## Supplementary Materials

Supplementary 1 Fig. S1. Photoreactivity analyses of NIR fluorophores. Fig. S2. Dose responses of thermogenesis and decomposition of cRGD-ZW800-PEG. Fig. S3. Phototherapeutic efficacy of cRGD-ZW800-PEG.

## References

[1] Wakiyama H, Kato T, Furusawa A, Choyke PL, Kobayashi H. Near infrared photoimmunotherapy of cancer; possible clinical applications. Nano. 2021;10(12):3135–3151. 10.1515/nanoph-2021-0119 PMC9646249 36405499

[2] Chow LQM. Head and neck cancer. N Engl J Med. 2020;382(1):60–72. 31893516 10.1056/NEJMra1715715

[3] Haddad RI, Shin DM. Recent advances in head and neck cancer. N Engl J Med. 2008;359(2):1143–1154. 18784104 10.1056/NEJMra0707975

[4] Argiris A, Karamouzis MV, Raben D, Ferris RL. Head and neck cancer. Lancet. 2008;371:1695–1709. 18486742 10.1016/S0140-6736(08)60728-X PMC7720415

[5] van Hinte G, Leijendekkers RA, Merkx MAW, Takes RP, Nijhuis-van der Sanden MWG, Speksnijder CM. Identifying unmet needs and limitations in physical health in survivors of head and neck cancer. Eur J Cancer Care (Engl). 2021;30(5): Article e13434. 33709466 10.1111/ecc.13434 PMC8519003

[6] Wu D, Zhao Z, Wang N, Zhang X, Yan H, Chen X, Fan Y, Liu W, Liu X. Fluorescence imaging-guided multifunctional liposomes for tumor-specific phototherapy for laryngeal carcinoma. Biomater Sci. 2020;8(12):3443–3453. 32412569 10.1039/d0bm00249f

[7] Wang X, Ramamurthy G, Shirke AA, Walker E, Mangadlao J, Wang Z, Wang Y, Shan L, Schluchter MD, Dong Z, et al. Photodynamic therapy is an effective adjuvant therapy for image-guided surgery in prostate cancer. Cancer Res. 2020;80(2):156–162. 31719100 10.1158/0008-5472.CAN-19-0201 PMC9641978

[8] Wang X, Sun R, Wang J, Li J, Walker E, Shirke A, Ramamurthy G, Shan L, Luo D, Carmon L, et al. A low molecular weight multifunctional theranostic molecule for the treatment of prostate cancer. Theranostics. 2022;12(5):2335–2350. 35265213 10.7150/thno.68715 PMC8899574

[9] Jo G, Kim EJ, Park MH, Hyun H. Tumor targeting with methotrexate-conjugated zwitterionic near-infrared fluorophore for precise photothermal therapy. Int J Mol Sci. 2022;23(22):14127. 36430604 10.3390/ijms232214127 PMC9697011

[10] Wei X, Zhang C, He S, Huang J, Huang J, Liew SS, Zeng Z, Pu K. A dual-locked activatable phototheranostic probe for biomarker-regulated photodynamic and photothermal cancer therapy. Angew Chem Int Ed Engl. 2022;61(26): Article e202202966. 35396786 10.1002/anie.202202966

[11] Choi HS, Gibbs SL, Lee JH, Kim SH, Ashitate Y, Liu F, Hyun H, Park G, Xie Y, Bae S, et al. Targeted zwitterionic near-infrared fluorophores for improved optical imaging. Nat Biotechnol. 2013;31(2):148–153. 23292608 10.1038/nbt.2468 PMC3568187

[12] Park GK, Lee JH, Levitz A, El Fakhri G, Hwang NS, Henary M, Choi HS. Lysosome-targeted bioprobes for sequential cell tracking from macroscopic to microscopic scales. Adv Mater. 2019;31(14): Article e1806216. 30740778 10.1002/adma.201806216 PMC6574216

[13] Kang H, Kang MW, Kashiwagi S, Choi HS. NIR fluorescence imaging and treatment for cancer immunotherapy. J Immunother Cancer. 2022;10(7):e004936. 35858710 10.1136/jitc-2022-004936 PMC9305898

[14] Muhanna N, Cui L, Chan H, Burgess L, Jin CS, MacDonald TD, Huynh E, Wang F, Chen J, Irish JC, et al. Multimodal image-guided surgical and photodynamic interventions in head and neck cancer: From primary tumor to metastatic drainage. Clin Cancer Res. 2016;22(4):961–970. 26463705 10.1158/1078-0432.CCR-15-1235

[15] Yin X, Cheng Y, Feng Y, Stiles WR, Park SH, Kang H, Choi HS. Phototheranostics for multifunctional treatment of cancer with fluorescence imaging. Adv Drug Deliv Rev. 2022;189: Article 114483. 35944585 10.1016/j.addr.2022.114483 PMC9860309

[16] Park MH, Jo G, Kim EJ, Jung JS, Hyun H. Tumor-targeted near-infrared fluorophore for fluorescence-guided phototherapy. Chem Commun (Camb). 2020;56(30):4180–4183. 32167112 10.1039/d0cc01366h

[17] Gong B, Shen Y, Li H, Li X, Huan X, Zhou J, Chen Y, Wu J, Li W. Thermo-responsive polymer encapsulated gold nanorods for single continuous wave laser-induced photodynamic/photothermal tumour therapy. J Nanobiotechnol. 2021;19(1):41. 10.1186/s12951-020-00754-8 PMC7869504 33557807

[18] Deng X, Shao Z, Zhao Y. Solutions to the drawbacks of Photothermal and photodynamic cancer therapy. Adv Sci (Weinh). 2021;8(3):2002504. 33552860 10.1002/advs.202002504 PMC7856884

[19] Sozmen F, Kucukoflaz M, Ergul M, Sahin Inan ZD. Nanoparticles with PDT and PTT synergistic properties working with dual NIR-light source simultaneously. RSC Adv. 2021;11(3):2383–2389.

[20] Bao K, Tully M, Cardenas K, Wang H, Srinivas S, Rho J, Jeon OH, Dinh J, Yokomizo S, McDonnell R, et al. Ultralow background near-infrared fluorophores with dual-channel intraoperative imaging capability. Adv Healthc Mater. 2023;12(12): Article e2203134. 36640372 10.1002/adhm.202203134 PMC10175134

[21] Yang C, Wang H, Yokomizo S, Hickey M, Chang H, Kang H, Fukuda T, Song MY, Lee SY, Park JW, et al. ZW800-PEG: A renal clearable zwitterionic near-infrared fluorophore for potential clinical translation. Angew Chem Int Ed Engl. 2021;60(25):13847–13852. 33857346 10.1002/anie.202102640 PMC8428668

[22] Samanta A, Vendrell M, Das R, Chang YT. Development of photostable near-infrared cyanine dyes. Chem Commun (Camb). 2010;46(39):7406–7408. 20830356 10.1039/c0cc02366c

[23] Li DH, Smith BD. Deuterated indocyanine green (ICG) with extended aqueous storage shelf-life: Chemical and clinical implications. Chemistry. 2021;27(58):14535–14542. 34403531 10.1002/chem.202102816 PMC8530945

[24] Wang H, Kang H, Dinh J, Yokomizo S, Stiles WR, Tully M, Cardenas K, Srinivas S, Ingerick J, Ahn S, et al. P800SO3-PEG: A renal clearable bone-targeted fluorophore for theranostic imaging. Biomater Res. 2022;26(1):51. 36183117 10.1186/s40824-022-00294-2 PMC9526902

[25] Liu Y, Teng L, Lou XF, Zhang XB, Song G. “Four-in-one” design of a hemicyanine-based modular scaffold for high-contrast activatable molecular afterglow imaging. J Am Chem Soc. 2023;145(9):5134–5144. 36823697 10.1021/jacs.2c11466

[26] Nani RR, Kelley JA, Ivanic J, Schnermann MJ. Reactive species involved in the regioselective photooxidation of heptamethine cyanines. Chem Sci. 2015;6(11):6556–6563. 26508998 10.1039/c5sc02396c PMC4618397

[27] Owens EA, Henary M, El Fakhri G, Choi HS. Tissue-specific near-infrared fluorescence imaging. Acc Chem Res. 2016;49(9):1731–1740. 27564418 10.1021/acs.accounts.6b00239 PMC5776714

[28] Sanchez-Barcelo EJ, Mediavilla MD. Recent patents on light based therapies: Photodynamic therapy, photothermal therapy and photoimmunotherapy. Recent Pat Endocr Metab Immune Drug Discov. 2014;8(1):1–8. 24372346 10.2174/1872214807666131229103707

[29] Rylova SN, Barnucz E, Fani M, Braun F, Werner M, Lassmann S, Maecke HR, Weber WA. Does imaging alphavbeta3 integrin expression with PET detect changes in angiogenesis during bevacizumab therapy? J Nucl Med. 2014;55(11):1878–1884. 25278514 10.2967/jnumed.114.137570

[30] Handgraaf HJM, Boonstra MC, Prevoo H, Kuil J, Bordo MW, Boogerd LSF, Sibinga Mulder BG, Sier CFM, Vinkenburg-van Slooten ML, Valentijn A, et al. Real-time near-infrared fluorescence imaging using cRGD-ZW800-1 for intraoperative visualization of multiple cancer types. Oncotarget. 2017;8(11):21054–21066. 28416744 10.18632/oncotarget.15486 PMC5400565

[31] Daghastanli NA, Itri R, Baptista MS. Singlet oxygen reacts with 2′,7′-dichlorodihydrofluorescein and contributes to the formation of 2′,7′-dichlorofluorescein. Photochem Photobiol. 2008;84(5):1238–1243. 18422880 10.1111/j.1751-1097.2008.00345.x

[32] Patsenker E, Stickel F. Role of integrins in fibrosing liver diseases. Am J Physiol Gastrointest Liver Physiol. 2011;301(3):G425–G434. 21659620 10.1152/ajpgi.00050.2011

[33] Terry SY, Abiraj K, Frielink C, van Dijk LK, Bussink J, Oyen WJ, Boerman OC. Imaging integrin alphavbeta3 on blood vessels with 111In-RGD2 in head and neck tumor xenografts. J Nucl Med. 2014;55(2):281 –286. 24408894 10.2967/jnumed.113.129668

[34] Hu Q, Huang Z, Duan Y, Fu Z, Bin L. Reprogramming tumor microenvironment with photothermal therapy. Bioconjug Chem. 2020;31(5):1268–1278. 32271563 10.1021/acs.bioconjchem.0c00135

[35] Gao W, Li S, Liu Z, Sun Y, Cao W, Tong L, Cui G, Tang B. Targeting and destroying tumor vasculature with a near-infrared laser-activated “nanobomb” for efficient tumor ablation. Biomaterials. 2017;139:1–11. 28578297 10.1016/j.biomaterials.2017.05.037

[36] Dewhirst MW, Viglianti BL, Lora-Michiels M, Hanson M, Hoopes PJ. Basic principles of thermal dosimetry and thermal thresholds for tissue damage from hyperthermia. Int J Hyperth. 2003;19(3):267–294. 10.1080/0265673031000119006 12745972

[37] Yarmolenko PS, Moon EJ, Landon C, Manzoor A, Hochman DW, Viglianti BL, Dewhirst MW. Thresholds for thermal damage to normal tissues: An update. Int J Hyperth. 2011;27(4):320–343. 10.3109/02656736.2010.534527 PMC3609720 21591897

[38] Chu KF, Dupuy DE. Thermal ablation of tumours: Biological mechanisms and advances in therapy. Nat Rev Cancer. 2014;14(3):199–208. 24561446 10.1038/nrc3672

[39] West CL, Doughty ACV, Liu K, Chen WR. Monitoring tissue temperature during photothermal therapy for cancer. J BioX Res. 2019;2(4):159–168. 33088609 10.1097/jbr.0000000000000050 PMC7575041

[40] Hamblin MR, Nelson ST, Strahan JR. Photobiomodulation and cancer: What is the truth? Photomed Laser Surg. 2018;36(5):241–245. 29466089 10.1089/pho.2017.4401 PMC5946726

[41] Cheng P, Pu K. Molecular imaging and disease theranostics with renal-clearable optical agents. Nat Rev Mater. 2021;6:1095–1113.

[42] Jiang Y, Huang J, Xu C, Pu K. Activatable polymer nanoagonist for second near-infrared photothermal immunotherapy of cancer. Nat Commun. 2021;12(1):742. 33531498 10.1038/s41467-021-21047-0 PMC7854754

