# Erratum to “Bifunctional Tumor-Targeted Bioprobe for Phototheranosis”

**DOI:** 10.34133/bmr.0036

**Published:** 2024-05-29

**Authors:** Hae Sang Park, Shinya Yokomizo, Haoran Wang, Sophia Manganiello, Hailey Monaco, Rose McDonnell, Hajin Joanne Kim, Jiyun Rho, Sung Ahn, Jason Gladstone, Harry Jung, Homan Kang, Kai Bao, Satoshi Kashiwagi, Hak Soo Choi

**Affiliations:** ^1^ Gordon Center for Medical Imaging, Department of Radiology, Massachusetts General Hospital and Harvard Medical School, Boston, MA 02114, USA.; ^2^ Department of Otorhinolaryngology-Head and Neck Surgery, College of Medicine, Hallym University, Chuncheon 24253, South Korea.; ^3^ Institute of New Frontier Research Team, Hallym Clinical and Translation Science Institute, Hallym University, Chuncheon 24252, South Korea.

In the Research Article "Bifunctional Tumor-Targeted Bioprobe for Phototheranosis" [1], the authors requested to change the term “phothotheranosis” to “phototheranosis”. “Phototheranosis” is a new technical term that was recently created to represent a new area of research in this field. The spelling has been corrected in the title and supplementary materials.

Jason Gladstone was also added as an author. The updated author list is:

Hae Sang Park, Shinya Yokomizo, Haoran Wang, Sophia Manganiello, Hailey Monaco, Rose McDonnell, Hajin Joanne Kim, Jiyun Rho, Sung Ahn, Jason Gladstone, Harry Jung, Homan Kang, Kai Bao, Satoshi Kashiwagi, Hak Soo Choi

The Author contributions were updated to:

H.S.P., H.M., S.M., R.M., H.J.K., and S.A. performed the NIR imaging and photon-induced therapy experiments. H.S.P. and H.J. performed in vivo ROS generation assay and histological evaluation. S.Y., H.W., S.A., J.G., H.K., and K.B. performed the chemical synthesis and characterization. S.Y., H.M., S.M., R.M., H.J.K., and J.R. performed cell-based assays. H.S.P., S.Y., H.W., S.M., H.M., R.M., H.J.K., J.G., S.K., and H.S.C. reviewed, analyzed, and interpreted the data. H.S.P., S.K., and H.S.C. wrote the manuscript. All authors discussed the results and commented on the manuscript.

These changes have now been made in the PDF and HTML (full text).
