# Supplementary material for: Bifunctional Tumor-Targeted Bioprobe for Phototheranosis
Source: Biomater Res. 2024 Jan 25;28:0002. doi: 10.34133/bmr.0002 (PMC10845606; doi:10.34133/bmr.0002)
Supplement: Supplementary 1 — Fig. S1. Photoreactivity analyses of NIR fluorophores. Fig. S2. Dose responses of thermogenesis and decomposition of cRGD-ZW800-PEG. Fig. S3. Phototherapeutic efficacy of cRGD-ZW800-PEG. [file bmr.0002.f1.docx]

**Supporting Information**

**Bifunctional tumor-targeted bioprobe for phothotheranosis**

Hae Sang Park^[a,b]^, Shinya Yokomizo^[a]^, Haoran Wang^[a]^, Sophia Manganiello^[a]^, Hailey Monaco^[a]^, Rose McDonnell^[a]^, Hajin Joanne Kim^[a]^, Jiyun Rho^[a]^, Sung Ahn^[a]^, Harry Jung^[c]^, Homan Kang^[a]^, Kai Bao^[a]^, Satoshi Kashiwagi^[a]^ and Hak Soo Choi^[a]^

^[a]^ Prof. H. S. Park, H. Wang, S. Yokomizo, S. Manganiello, H. Monaco, R. McDonnell, H. J. Kim, J. Rho, S. Ahn, Prof. H Kang, Prof. K Bao, Prof. S. Kashiwagi, Prof. H. S. Choi

Gordon Center for Medical Imaging, Department of Radiology, Massachusetts General Hospital and Harvard Medical School, Boston, MA 02114, USA

E-mail: hchoi12@mgh.harvard.edu, skashiwagi@ mgh.harvard.edu

^[b]^ Prof. H. S. Park

Department of Otorhinolaryngology-Head and Neck Surgery, College of Medicine, Hallym University, Chuncheon, 24253, South Korea

^[c]^ H. Jung

Institute of New Frontier Research Team, Hallym Clinical and Translation Science Institute, Hallym University, Chuncheon, 24252, South Korea

*Corresponding Authors: hchoi12@mgh.harvard.edu, skashiwagi@ mgh.harvard.edu

**Table of Contents**

**Figure S1.** Photoreactivity analyses of NIR fluorophores.

**Figure S2.** Dose responses of thermogenesis and decomposition of cRGD-ZW800-PEG.

**Figure S3**. Phototherapeutic efficacy of cRGD-ZW800-PEG.


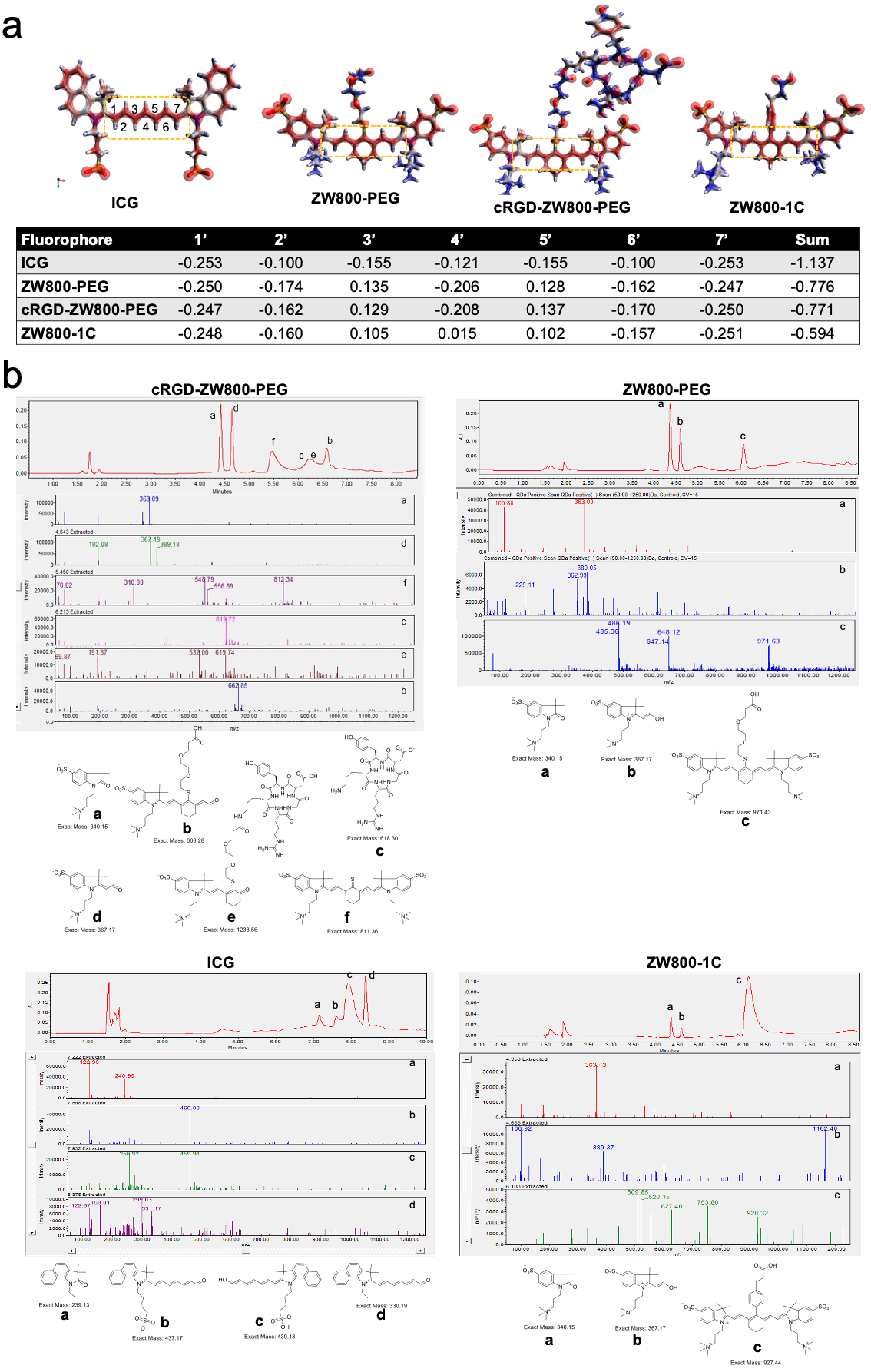


**Figure S1. Photoreactivity analyses of NIR fluorophores.** (a) Electron density of cRGD-ZW800-PEG. (b) Fragmentation analysis of the selected NIR fluorophore upon the laser irradiations. 20-50 µM working solutions were prepared in PBS from 10 mM stock solutions in DMSO (> 97% purity). Then the solution was irradiated with an 808 nm laser at a power of 1 W/cm^2^ for 2-5 min until the fade of green color. After laser irradiation, the sample solution was applied to HPLC-MS analysis.


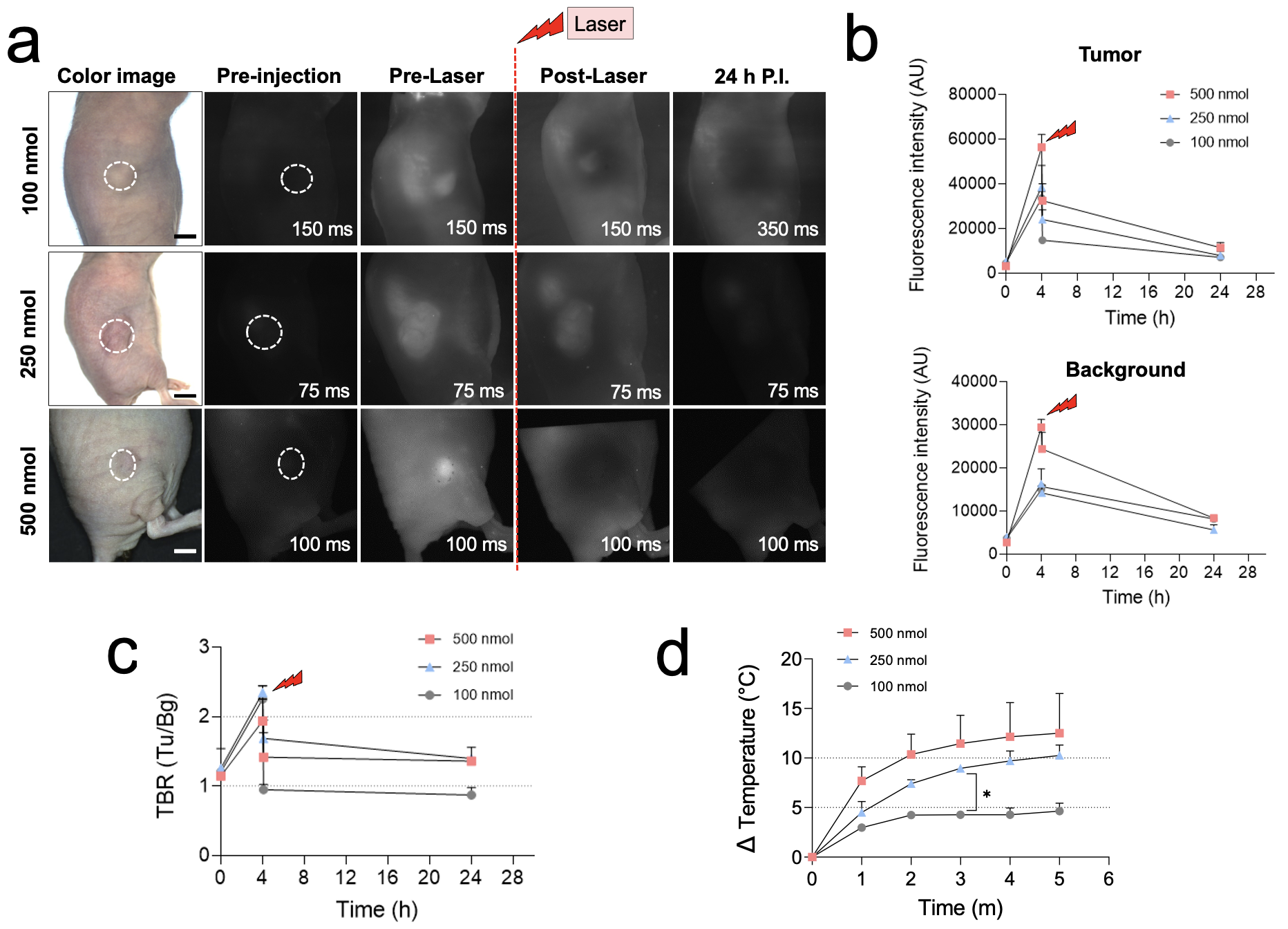
**Figure S2. Dose responses of thermogenesis and decomposition of cRGD-ZW800-PEG under 808 nm laser irradiation.** (a) Representative time-course NIR images during the laser treatment. NIR fluorescence imaging was conducted using the FLARE imaging system at pre-injection, pre-laser (4 h post-injection of cRGD-ZW800-PEG), post-laser (immediate after laser irradiation), and post-24 h injection (24 h P.I.) of cRGD-ZW800-PEG. Scale bar = 5 mm. (b) Quantitative time-course assessment of fluorescence intensity (AU) of tumor and background. (c) Quantitative time-course assessment of TBR. Fluorescence intensity and TBR were observed at pre-injection, pre-laser (4 h post-injection of cRGD-ZW800-PEG), post-laser (immediate after laser irradiation), and post-24 h injection of cRGD-ZW800-PEG by NIR imaging (*n* = 2 in 500 and 250 nmol, *n* = 3 in 100 nmol, mean ± s.e.m.). (d) Temperature changes of the irradiated tumor (*n* = 2 in 500 and 250 nmol, *n* = 3 in 100 nmol, mean ± s.e.m.). **P* < 0.05 by two-way ANOVA followed by Tukey’s multiple comparison tests.

**
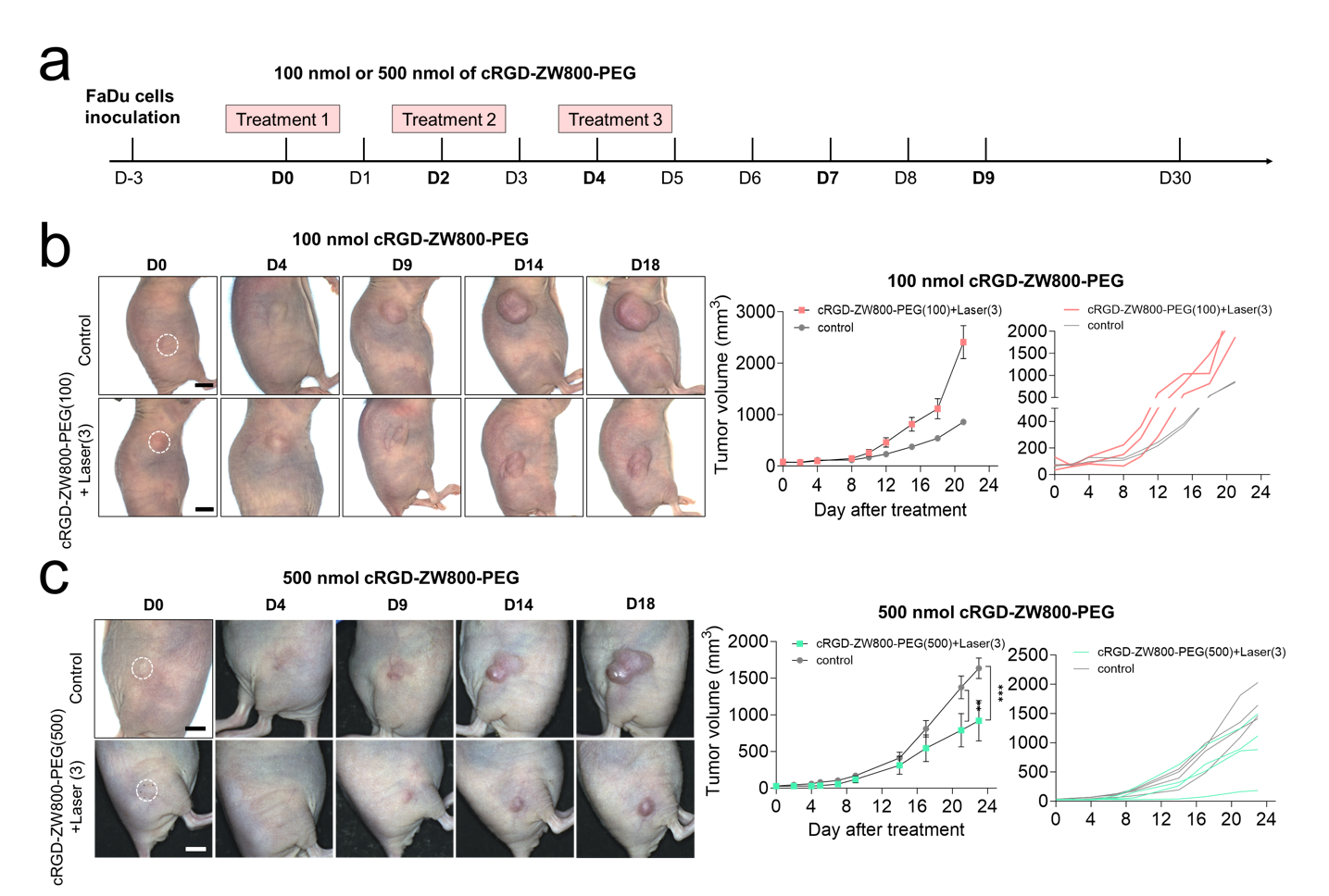
**

**Figure S3. Phototherapeutic efficacy of cRGD-ZW800-PEG.** (a) A schematic of the laser treatment. Tumors were irradiated by an 808 nm laser (1.0 W/cm^2^) for 5 min 4 h post-injection of cRGD-ZW800-PEG in tumor-bearing mice. A total of 3 times of laser irradiation was performed on the same site at intervals of 2 d. (b) Representative photos and growth curves (average and individual) of the xenograft tumor in the mouse back over 18 d after the initiation of the treatments. The control group indicates no treatment (no fluorophore and no laser) (*n* = 2, mean ± s.e.m). Tumor-bearing mice were injected with 100 nmol cRGD-ZW800-PEG followed by irradiation with an 808 nm laser at 1.0 W/cm^2^ for 5 min every 2 d (*n* =3, mean ± s.e.m), Scale bar = 5 mm. (c) Representative photos and growth curves (average and individual) of the tumor of the xenograft tumor over 30 d after the initial treatment. The control group indicates no treatment (no fluorophore and no laser) (*n* = 4, mean ± s.e.m). Tumor-bearing mice were injected with 500 nmol cRGD-ZW800-PEG followed by irradiation with an 808 nm laser at 1.0 W/cm^2^ for 5 min every 2 d (3 times in total) (*n* = 4, mean ± s.e.m). Scale bar = 5 mm. ***P* < 0.01, ****P* < 0.001 by two-way ANOVA followed by Šídák's multiple comparison tests.
